# Distress tolerance as a mechanism of mindfulness for depression and anxiety: Cross-sectional and diary evidence

**DOI:** 10.1016/j.ijchp.2023.100392

**Published:** 2023-07-05

**Authors:** Yanjuan Li, Ruilin Ju, Stefan G. Hofmann, Wingsze Chiu, Ye Guan, Yu Leng, Xinghua Liu

**Affiliations:** a Beijing Key Laboratory of Behavior and Mental Health, School of Psychological and Cognitive Sciences, Peking University, Beijing, China; b Department of Clinical Psychology, Philipps University Marburg, Germany

**Keywords:** Mindfulness, Distress tolerance, Depression, Anxiety, Mechanism, Daily diary

## Abstract

**Background:**

Both trait and state mindfulness are associated with less depression and anxiety, but the mechanisms remain unknown. Distress tolerance, an important transdiagnostic factor of emotional disorders, may mediate the relationship between mindfulness and depression/anxiety.

**Method:**

Study 1 examined the mediation model at the between-person level in a large cross-sectional sample (*n* = 905). In Study 2, a daily diary study (*n* = 110) was conducted to examine within-person changes. Participants were invited to complete daily diaries measuring daily mindfulness, distress tolerance, depression and anxiety for 14 consecutive days.

**Results:**

In Study 1, results of simple mediation analyses indicated that distress tolerance mediated the relationship between mindfulness and depression/anxiety at the between-person level. In Study 2, results of multilevel mediation analyses indicated that, in both the concurrent model and time-lagged model, daily distress tolerance mediated the effects of daily mindfulness on daily depression/anxiety at both the within- and between-person level.

**Conclusions:**

Distress tolerance is a mechanism underlying the relationship between mindfulness and depression/anxiety. Individuals with high or fluctuating depression and anxiety may benefit from short-term or long-term mindfulness training to increase distress tolerance.

Mindfulness requires the person to intentionally pay attention to the present internal and external experiences in a nonjudgmental and accepting manner (Bishop et al., 2004). It is usually conceptualized at a trait level (relatively stable between-person differences in the tendency to be mindful in daily life) or at a state level (within-person fluctuations in the engagement of the mindfulness process; e.g., Eisenlohr-Moul et al., 2016; Germer et al., 2005). Mindfulness has been consistently shown to have an inverse relationship with depression and anxiety, as demonstrated by cross-sectional studies (Barcaccia et al., 2019; Desrosiers et al., 2013; Prieto-Fidalgo et al., 2022), intensive longitudinal studies (e.g., Enkema et al., 2020), and randomized controlled studies (e.g., Taylor et al., 2021). The mechanisms through which mindfulness improves mental health have been proposed by different theoretical models, such as exposure, decentering, emotional and cognitive flexibility, and nonattachment (Baer, 2003; Brown et al., 2007; Shapiro et al., 2006). However, there is still a need for empirical studies to test these mechanisms, especially exposure and related processes/abilities that have been proposed by most researchers.

Distress tolerance, the perceived or actual ability to withstand negative experiential emotions, plays a critical role in exposure and can be developed through repeated practice (Allen et al., 2007; Craske et al., 2008; Lynch & Mizon, 2011). Research has suggested that individuals high in mindfulness have greater distress tolerance due to their ability to respond to aversive tension rather than react automatically with avoidance or escape (Lynch & Mizon, 2011). Trait mindfulness has been found to be positively associated with distress tolerance (Brem et al., 2019; Elhai et al., 2018), and even brief meditation training that induces state mindfulness can increase individuals’ distress tolerance (Carpenter et al., 2019; Sauer & Baer, 2010). Long-term MBIs have been found to increase distress tolerance in both clinical and nonclinical samples, and increases in state and trait mindfulness have been found to predict increases in distress tolerance (Kraemer et al., 2020; Lotan et al., 2013).

Distress tolerance is an important transdiagnostic factor underlying anxiety disorders and depression (For a review, see Leyro et al., 2010). Low distress tolerance is characterized by a heightened sensitivity to negative emotions, which are perceived as intolerable and avoided through suppression or emotion-driven behaviors (e.g., Leyro et al., 2010; Simons & Gaher, 2005; Trafton & Gifford, 2011). Although these strategies may provide short-term relief, they can exacerbate emotional distress in the long run (Barlow et al., 2010; Ellard et al., 2010; Simons & Gaher, 2005). Evidence from cross-sectional (Lass & Winer, 2020; Reitzel et al., 2017; Yoon et al., 2018) and longitudinal studies (Hashoul-Andary et al., 2015; Lin et al., 2018) suggests that low distress tolerance is associated with increased depression and anxiety.

Although distress tolerance is typically construed as a relatively stable personality characteristic, it can also fluctuate over time within an individual (Hawkins et al., 2013; Veilleux et al., 2018) following a dynamic trajectory (Trafton & Gifford, 2011). Using a daily diary study design, Hawkins et al. (2013) observed that daily distress tolerance was negatively associated with daily negative affect. Similar effects were reported when examining distress tolerance using high-density (7 times per day) data assessments (Veilleux et al., 2018). In line with the literature, this study found that momentary distress tolerance was inversely related to momentary negative affect.

Based on previous research, distress tolerance may mediate the relationship between mindfulness and depression/anxiety (Brem et al., 2019; Leyro et al., 2010; Lynch & Mizon, 2011). However, direct examination of distress tolerance as a mediator of mindfulness is lacking (Baer, 2003). Considering the trait-like and state-like attributes of these constructs, investigating their relationships at both between- and within-person levels is crucial. Cross-sectional studies can offer preliminary insights into between-person relationships, while daily diary studies with repeated measurements can capture within-person relationships (Bolger & Laurenceau, 2013). Concurrent and time-lagged models can be used to explore potentially causal associations or temporal precedence among variables (e.g., Felsman et al., 2017).

Above all, the current study aimed to investigate the mediating effect of distress tolerance using both a large sample cross-sectional design (Study 1) and a daily diary study design (Study 2). Our hypothesis for Study 1 was that at the between-person level, distress tolerance played a mediating role between trait mindfulness and depression/anxiety. Our hypothesis for Study 2 was that at the within-person level, daily distress tolerance mediated the relationship between daily mindfulness and depression/anxiety. Apart from their concurrent relationships, Study 2 also examined the indirect effects of distress tolerance when variables were lagged in the order specified by the model. The time-lagged relationships were examined exploratively.

## Study 1

In Study 1, we examined the relationship between mindfulness, distress tolerance, and depression/anxiety in a large sample of adults. The relatively large sample allowed us to examine the generalizability of the model, in which distress tolerance mediated the relationship between mindfulness and depression/anxiety at the between-person level.

### Methods

#### Participants

The current study used baseline data drawn from a study examining the effects of the self-help Mindfulness Intervention for Emotional distress (MIED) program ((http://www.chictr.org.cn/, Registration number: ChiCTR2000034193 ). Inclusion criteria included : (1) aged 18 to 65, and (2) with at least moderate emotional distress (score of the 10-item Kessler Psychological Distress Scale ≥ 22; K10; Kessler et al., 2002). Exclusion criteria included (1) no access to the Internet and (2) inadequate proficiency in Chinese.

In total, 1056 participants finished the baseline measures, of whom 147 were excluded for more than 1 incorrect answer on the screening questions (e.g., “*Please choose ‘never’ on this item*”), and 4 participants were excluded as they were under the age of 18, leaving a final sample size of 905. The final sample consisted of mostly women (*n* = 736; 81.32%), with an average age of 32.00 (*SD* = 9.69) and an average length of education of 16.22 (SD = 2.65). Among them, 28.29% were full-time students; 12.60% were teachers; 9.39% were professionals (e.g., lawyers and medical workers); and 49.72% were employees with other jobs.

#### Procedure

The Association for Ethics and Human and Animal Protection of the School of Psychological and Cognitive Sciences of Peking University approved the current study. Participants were recruited from July to December 2020 using various social media platforms. Specifically, recruitment efforts were conducted on our WeChat Official Account, where a targeted advertisement was posted. The advertisement included a concise study description and prompted potential participants to complete a sign-up questionnaire.

Participants who met the inclusion criteria but not the exclusion criteria were further invited to complete a baseline questionnaire. After that, Participants were provided with a self-help MIED program (Liu, in press) and invited to complete weekly questionnaires, but only data from baseline measures were used in the current study.

#### Measures

*Mindfulness* The 20-item Five-Facet Mindfulness Questionnaire-Short Form (FFMQ-SF; Hou et al., 2014) was used to measure mindfulness. The FFMQ-SF has five dimensions: observing, describing, acting with awareness, nonjudgement, and nonreactivity. The hierarchical five-factor models of FFMQ-SF demonstrated good model fit (Hou et al., 2014). An example item is “I find myself doing things without paying attention.” Items were scored on a five-point Likert scale ranging from 1 (never or very rarely true) to 5 (often or always true). The higher the total score on the scale, the higher the level of mindfulness. In the current study, the Cronbach's α of FFMQ-SF was .750.

*Distress tolerance* The 15-item Distress Tolerance Scale (DTS; Simon & Gaher, 2005) was used to assess distress tolerance. It contains four dimensions: regulation, absorption, appraisal, and tolerance. Simons and Gaher (2005) identified both a single second-order general distress tolerance factor and four first-order factors as the final structure of the scale. An example item is “I'll do anything to avoid feeling distressed or upset”. Items were scored on a five-point Likert scale ranging from 1 (strongly agree) to 5 (strongly disagree). The higher the total score on the scale, the higher the level of distress tolerance. In the current study, the Cronbach's α of DTS was .897.

*Anxiety* Overall Anxiety Severity and Impairment Scale (OASIS; Norman et al., 2011) was used to measure anxiety. It contains 5 items, which were scored on a five-point Likert scale ranging from 0 to 4. For example, one question is “How often do you feel anxious? (Never-Constantly)”. Items were summed to obtain one total score. The higher the score, the more severe and impaired the anxiety is. In the current study, the Cronbach's α of OASIS was .869.

*Depression* Overall Depression Severity and Impairment Scale (ODSIS; Bentley et al., 2014) was used to measure depression. Like the OASIS, the ODSIS contains 5 items, coded from 0 to 4, and summed to obtain one total score. One question, for instance, is “How much has depression interfered with your social life and relationships? (Not at all - Extreme)”. In the current study, the Cronbach's α of ODSIS was .928.

#### Data analyses

Preliminary analyses and mediation analysis were conducted using SPSS 26.0 and Mplus 7.4 respectively.

Preliminary analyses included examining the relationships between demographic variables and anxiety/depression and assessing the correlations among the study variables. For mediation analysis, a bootstrapping approach with 2000 bootstrap samples was implemented to obtain 95% bias-corrected confidence intervals (CI) for the indirect effect (Preacher & Hayes, 2004). If the 95% CI does not contain zero, the indirect effect will be considered significant. Based on the criteria proposed by Hu and Bentler (1999), CFI > 0.90, RMSEA < 0.08, and SRMR < 0.1 suggest a good fit for the model. We also reported Akaike's information criterion (AIC), and the Bayesian information criterion (BIC), which reflected a penalty for the number for parameters in the model.

In addition, we explored an alternative mediation model where distress tolerance was treated as the predictor variable and mindfulness as the mediator variable, reversing the order of the variables. To compare these two models, we primarily relied on the model fit indices of AIC and BIC. According to the criterion of smaller-is-better, we considered differences of 2 or more in AIC and BIC values as meaningful indicators of differences between the models (Kass & Raftery, 1995).

### Results

#### Preliminary analyses

No gender differences existed among all study variables (all *p*-values > .05). As shown in Table 1, Age was correlated with anxiety but not with depression. Length of education was not correlated with the dependent variable. Therefore, only age was controlled in the mediation model.

**Table 1:** Correlations between mindfulness, distress tolerance, depression and anxiety.

	1	2	3	4	5
1 Age					
1 Length of education	−.103⁎⁎				
1 Mindfulness	.127⁎⁎⁎	.054			
1 Distress tolerance	.080*	.007	.433⁎⁎⁎		
1 Anxiety	−.117⁎⁎⁎	−.011	−.449⁎⁎⁎	−.527⁎⁎⁎	
1 Depression	−.059	−.044	−.384⁎⁎⁎	−.476⁎⁎⁎	.750⁎⁎⁎

Note.

⁎⁎⁎ *p* < .001.

⁎⁎ *p* < .01.

⁎ *p* < .05.

Mindfulness, distress tolerance, depression and anxiety were significantly correlated with each other (correlations ranged from -.384 to -0.750, all *p*-values > .05; see Table 1), indicating moderate to strong negative associations among these variables.

#### Mediation model

**Fig 1:**
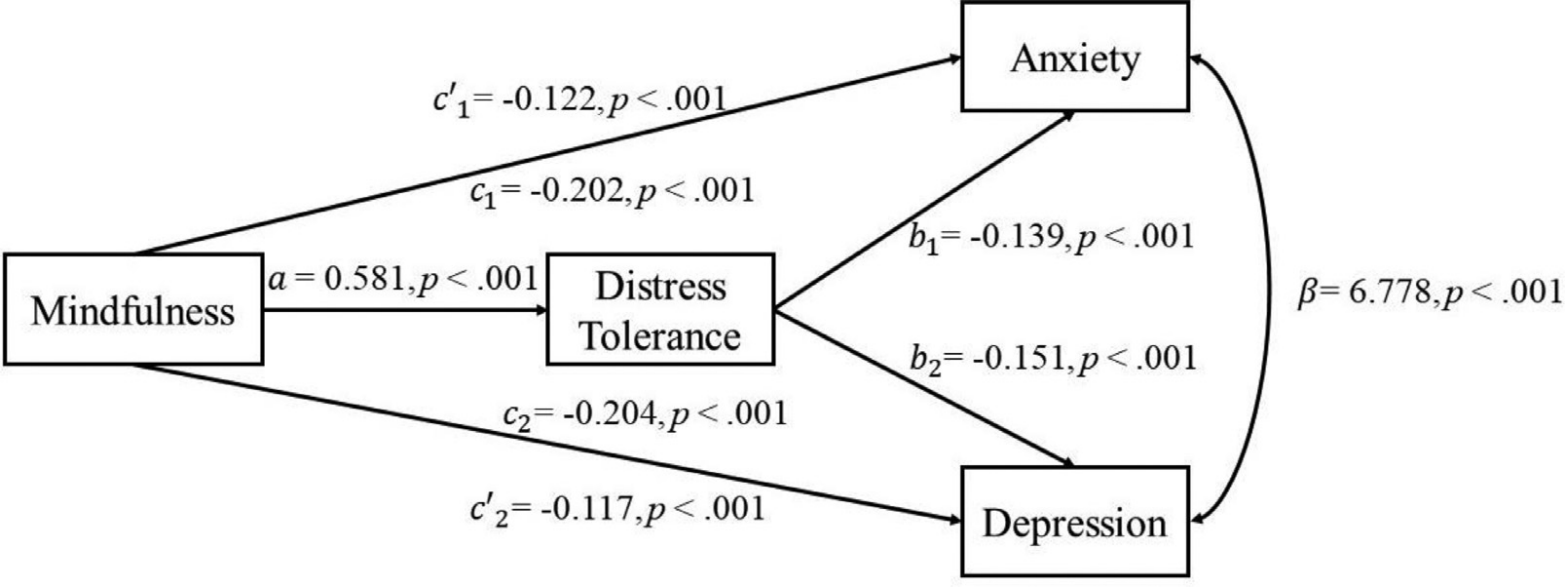
Path diagram of distress tolerance mediating the relationship between mindfulness and depression/anxiety of Study 1.

As shown in Fig 1, after controlling age, mindfulness positively predicted distress tolerance (*a* = 0.581, *p* < .001) and negatively predicted anxiety (*c*’_1_ = -0.122, *p* < .001) /depression (*c*’_2_ = -0.117, *p* < .001), and distress tolerance negatively predicted anxiety (*b*_1_ = -0.139, *p* <.001)/depression (*b*_2_ = -0.151, *p* <.001). In addition, the paths from mindfulness through distress tolerance to both anxiety and depression were statistically significant (see Table 2). The model fit the data well (AIC = 21861, BIC = 21933, $\chi_{\left(3\right)}^{2}$ = 15.477, *p* = .002, CFI = 0.991, RMSEA = 0.068, 90%CI = [0.037, 0.103], SRMR = 0.039). Therefore, distress tolerance partially mediated the effects of mindfulness on both depression and anxiety.

**Table 2:** Indirect effects of the mediation model of Study 1.

	**Indirect Effects**	***SE***	**95% CI**	**Effect size**
a*b_1_	−0.081	0.008	[-0.098, -0.066]	40.10%
a*b_2_	−0.088	0.009	[-0.105 -0.070]	43.14%

*Note. SE* = standard error, CI = confidence interval. Effect size = the ratio of the indirect effect to the total effect×100%.

When considering the reversed mediation model with distress tolerance as the predictor and mindfulness as the mediator, we found significant indirect effects (refer to Supplementary Materials for Study 1, Fig S1). However, the model fit indices, specifically AIC and BIC, were larger for the reversed mediation model (AIC = 22025, BIC = 22102). Therefore, we preferred the model in which distress tolerance was set as the mediator.

### Study 1: Summary of the results

Using a large sample cross-sectional study, Study 1 investigated the mediating effect of distress tolerance on mindfulness's association with depression/anxiety at the between-person level. Consistent with our hypotheses, the results of Study 1 revealed that individuals with higher levels of mindfulness demonstrated greater distress tolerance, which, in turn, was associated with lower levels of depression and anxiety. These findings provide preliminary support for the proposed mediation model and align with previous theoretical frameworks highlighting exposure as a mechanism of mindfulness (e.g., Baer et al., 2003; Brown et al., 2007; Shapiro et al., 2006).

By investigating the interplay between mindfulness, distress tolerance, and emotional distress, Study 1 contributed to the understanding of potential mechanisms underlying the beneficial effects of mindfulness. However, it is important to note that the cross-sectional nature of the study limits our ability to draw causal conclusions and examine the within-person relationships.

## Study 2

To replicate the results of Study 1 and to further examine the mediating effects of distress tolerance in the relationship between mindfulness and depression/anxiety at the within-person level, we conducted Study 2. This study is a daily dairy study with more ecological validity. Apart from the concurrent model, we also planned to establish a time-lagged model to examine the temporal precedence of daily mindfulness, distress tolerance, and depression/anxiety. In this way, we could investigate whether distress tolerance met the requirement of temporal precedence for playing a mechanical role (Kazdin, 2007).

### Methods

#### Participants

One hundred and thirty-four participants with at least moderate emotional distress (K10 ≥ 22; Kessler et al., 2002) were recruited. In total, 1440 daily diaries from 128 participants were received. Among them, 18 participants did not provide at least half of the daily measures (*n* < 7), and they were excluded from further analyses, leading to a final sample size of 110 (82.84%). No differences were found in all baseline measures between those who were excluded and those who were included in the current study (all *p*-values > .05).

The final sample consisted of mostly women (*n* = 93; 84.54%), with an average age of 32.15 years old (*SD* = 9.89). The average length of education was 16.45 years (SD = 2.62). Among them, 24.5% were full-time students;10.9% were teachers; 10.0% were professionals (e.g., lawyers and medical workers); and 54.6% were employees with other jobs.

#### Procedure

The study was approved by the Association for Ethics and Human and Animal Protection of [institution masked for review]. Participants were recruited through our WeChat Official Account in December, 2021, and each participant was required to complete a sign-up questionnaire attached to the advertisement. Among the respondents, those who met the eligibility criteria were selected and invited to join a dedicated WeChat Group.

In the WeChat Group, participants were instructed to complete daily measures, which remained consistent throughout the study. These measures were sent by the experimenter every day at 20:00. Participants were given a deadline of 24:00 each day to complete the measures. Upon the successful completion of the study, participants received online self-help MIED ( Liu, in press) as compensation for their participation.

##### Daily measures

All daily measures were adapted from the measures described in Study 1. Detailed descriptions are as follows.

##### Daily mindfulness

Based on Baer and colleagues’ study (2006), items with the highest factor from each subscale of the Five-Facet Mindfulness Questionnaire were chosen to form the Daily Mindfulness Scale (DMS). This resulted in five final items. An example item is “I perceive my feelings and emotions without having to react to them”. Participants were instructed to answer the questions based on their experience “today”. Response options ranged from 1 (strongly disagree) to 5 (strongly agree). The Intra-class correlation (ICC) of DMS is .497, suggesting that about half of the variance existed at the within-person level.

##### Daily distress tolerance

Based on a preliminary study, the items with the highest factor loading from each subscale of the DTS (Simon & Gaher, 2005) were chosen to form the Daily Distress Tolerance Scale (DDTS). This resulted in four items. An example item is “I can't handle feeling distressed or upset”. Participants were instructed to answer the questions based on their experience “today”. Items were scored on a five-point Likert scale ranging from 1 (strongly agree) to 5 (strongly disagree). The ICC of DDTS was .520.

##### Daily anxiety

Items from OASIS were adapted to form Daily Anxiety and Impairment Scale (DASIS; Norman et al., 2011). DASIS also contains five items, coded from 0 to 4. For example, one item is, “Today, how often do you feel anxious? (Never-Constantly)”. The ICC of DASIS was .462.

##### Daily depression

Items from ODSIS were adapted to form Daily Depression and Impairment Scale (DDSIS; Bentley et al., 2014). DDSIS also contains five items, coded from 0 to 4. For instance, one item is, “Today, how often do you feel depressed? (Never-Constantly)”. The ICC of DDSIS was .588.

### Data analyses

The preliminary analyses involved examining the correlations between demographic variables and daily anxiety/depression, as well as the correlations among the outcome variables. These analyses were conducted using SPSS version 26.0.

For daily measures (daily mindfulness-DMF; daily distress tolerance-DDT; daily anxiety-DA; daily depression-DD), given the nested data structure (daily measures nested within persons), multilevel structural equation modeling (MSEM; Preacher et al., 2011) was conducted. The MSEM distinguished between within-person and between-person components and explored the relationship between mindfulness and depression/anxiety at both levels, as well as the potential mediating role of distress tolerance. Based on the criteria proposed by Hu and Bentler (1999), CFI > 0.90, RMSEA < 0.08, and SRMR < 0.1 suggest a good fit for the model.

Both concurrent models (e.g., DMF_t_ → DDT_t_ → DA_t_/DD_t_) and time-lagged models (e.g., DMF_t-1_ → DDT_t_ → DA_t+1_/DD_t+1_) were tested. “t” denotes time (i.e., a certain day); “t-1” denotes the previous timepoint (i.e., the previous day); and “t+1” denotes the next timepoint (i.e., the next day). In addition, reversed multilevel mediation models with DDT as the predictor and DMF as the mediator were also analyzed (see Supplementary Materials). In this way, we could provide more information on the mechanical role of distress tolerance in the relationship between mindfulness and depression/anxiety.

The Monte Carlo method was adopted to examine the multilevel mediation effects. The number of replications was set to 2000, and the 95% Monte Carlo confidence intervals (MCCIs) were used to assess the indirect effects (Preacher & Selig, 2012). If the MCCI does not include zero, the indirect effect will be considered significant.

### Results

#### Preliminary analyses

Only participants provided at least half of the daily measures (n ≥ 7) were included in the analyses. In total, the final sample (*n* = 110) provided 1355 valid daily diaries (*M* = 12.32, *SD* = 1.76, range = [7,14]). We calculate the average number of days between different assessments by dividing the total number of days participants were involved in the study by the number of assessments completed. Based on our data, the average duration between consecutive assessments was approximately 1.14 days.

There were no gender differences in depression/anxiety (all *p*-values > .05). Length of education was not correlated with daily anxiety or daily depression (all *p*-values > .05). Age was marginally significantly and negatively correlated with daily anxiety (*r* = -.056, *p* = .055). Therefore, only age was included as a control variable in the following mediation analyses.

As shown in Table 3, daily mindfulness, daily distress tolerance, and daily depression/anxiety were significantly correlated with each other at the within- and between-person level (correlations ranged from -.182 to -.380 at the within-person level, ranged from -.238 to -.501 at the between-person level, all *p*-values > .05).

**Table 3:** Correlations between daily measures.

	1	2	3	4	5	6
1.Age						
2.Edu	−.121					
3.DMF	.053⁎⁎	.006		.193⁎⁎⁎	−.208⁎⁎⁎	−.182⁎⁎⁎
4.DDT	.011	.052	.472⁎⁎⁎		−.282⁎⁎⁎	−.287⁎⁎⁎
5.DA	−.056	.054	−.329⁎⁎⁎	−.411⁎⁎⁎		.380⁎⁎⁎
6.DD	−.050	−.015	−.238⁎⁎⁎	−.384⁎⁎⁎	.501⁎⁎⁎	

Note.

⁎⁎⁎ *p* < .001,

⁎⁎ *p* < .01, *p < .05; Within-person correlations are above the diagonal and between-person correlations are below the diagonal. DMF = daily mindfulness; DDT = daily distress tolerance; DA = daily anxiety; DD = daily depression.

#### Multi-level mediation models

**Fig 2:**
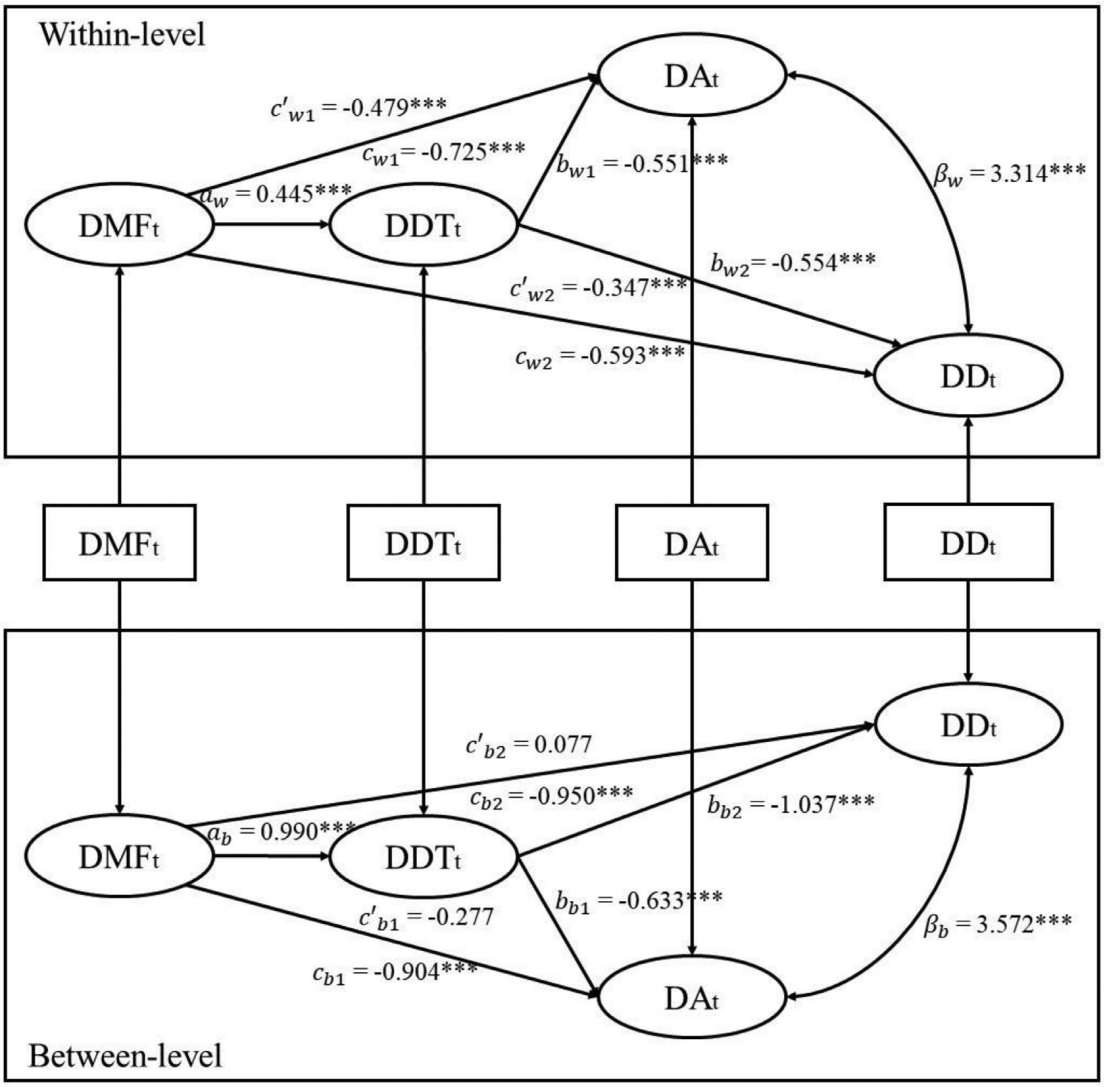
MSEM results for the 1-1-1 concurrent mediation model of DMF to DA/DD via DDT. ^⁎⁎⁎^*p* < .001, ^⁎⁎^*p* < .01, *p < .05; DMF = daily mindfulness;DDT = daily distress tolerance; DA = daily anxiety; DD = daily depression.

As shown in Fig 2, daily mindfulness negatively predicted depression/anxiety and positively predicted distress tolerance at both levels, while distress tolerance negatively predicted depression/anxiety at both levels. Analyses with the Monte Carlo method revealed that the 95% confidence intervals did not contain zero. These results supported the indirect effects of distress tolerance at both levels (See Table 4). The model fit the data well (AIC = 25486, BIC = 25622, $\chi_{\left(2\right)}^{2}$ = 16.053, *p* < .001, CFI = 0.986, RMSEA = 0.072, within-level SRMR = 0, between-level SRMR = 0.074). Therefore, distress tolerance partially mediated the effects of daily mindfulness on depression/anxiety at the within-person level, and fully mediated the effects of daily mindfulness on depression/anxiety at the between-person level.

**Table 4:** Indirect effects of the multilevel mediation model.

		**Indirect Effects**	***SE***	**95% MCCI**
**DMF_t_-DDT_t_-DA_t_/DD_t_**				
Within-level	a_w_*b_w__1_	−0.245	0.032	[-0.310, -0.186]
	a_w_*b_w__2_	−0.247	0.035	[-0.320, -0.181]
Between-level	a_b_*b_w__1_	−0.627	0.216	[-1.073, -0.230]
	a_w_*b_w__2_	−1.027	0.302	[-1.680, -0.484]
**DMF_t-1_-DDT_t_-DA_t+1_/DD _t+1_**				
Within-level	a_w_*b_w__1_	−0.013	0.008	[-0.034, -0.001]
	a_w_*b_w__2_	−0.011	0.008	[-0.032, -0.0003]
Between-level	a_b_*b_w__1_	−0.727	0.251	[-1.244, -0.264]
	a_w_*b_w__2_	−1.186	0.356	[-1.934, -0.542]

*Note. SE* = standard error, MCCI = Monte Carlo Confidence Interval. DMF = daily mindfulness;DDT = daily distress tolerance; DA = daily anxiety; DD = daily depression.

**Fig 3:**
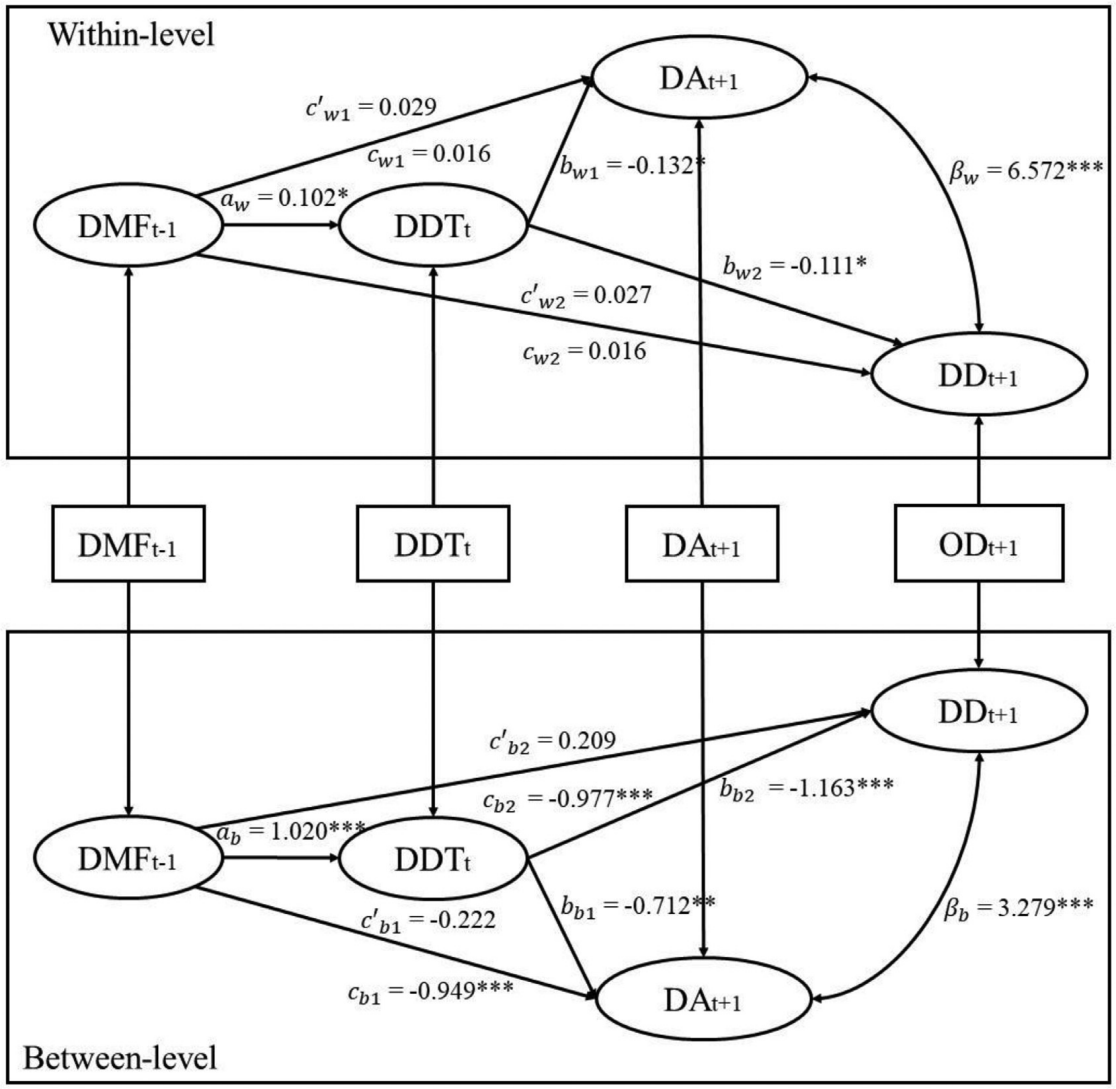
MSEM results for the 1-1-1 time-lagged mediation model of DMF at t-1 to DA/DD at t+1 via DDT at t. ^⁎⁎⁎^*p* < .001, ^⁎⁎^*p* < .01, *p < .05; DMF = daily mindfulness;DDT = daily distress tolerance; DA = daily anxiety; DD = daily depression.

The 1-1-1 time-lagged mediation model was also conducted and yielded similar results. The model fit the data well (AIC = 24874, BIC = 25012, $\chi_{\left(2\right)}^{2}$ = 15.713, *p* < .001, CFI = 0.976, RMSEA = 0.067, within-level SRMR= 0, between-level SRMR = 0.075). At both levels, distress tolerance at time t mediated the effects of daily mindfulness at time t-1 on depression/anxiety at time t+1 (see Fig 3 and Table 4), further supporting the mechanical role of distress tolerance.

We also tested alternative lagged mediation models, reversing the order of mindfulness and distress tolerance, to examine the specificity of temporal ordering in the mediation model. The mediation effect of mindfulness was only significant at the within-person level but not at the between-person level in both concurrent and the time-lagged models (details can be found in the Supplementary Materials for Study 2). Therefore, additional evidence was obtained supporting distress tolerance as the mediator of mindfulness for anxiety and depression.

### Study 2: Summary of the results

The present fourteen-day daily diary study was the first to demonstrate the unique contribution of day-to-day within-person variability in mindfulness to daily distress tolerance and depression/anxiety. More importantly, both concurrent and time-lagged mediation models supported the mediating effect of distress tolerance in the relationship between mindfulness and depression/anxiety at both between- and within-person levels. This showed that on days individuals were more mindful than usual, they could tolerate distress better and could experience less anxiety and depression. The temporal precedence of these variables was established by the lagged model and alternative temporal models in reverse order. In addition, the current study had a higher ecological validity by assessing mindfulness, distress tolerance, and depression/anxiety within a shorter time frame (i.e., daily), and repeatedly validated Study 1’s results. Based on the results of Study 2, distress tolerance as the mechanism underlying mindfulness also met the requirement of temporal precedence (Kazdin, 2007).

## General discussion

The two studies presented in this paper systematically examined whether distress tolerance was the underlying mechanism of the relationship between mindfulness and depression/anxiety from both trait and state perspectives. Study 1, using a large sample cross-sectional study, provided support for the mediating effect of distress tolerance at the between-person level. Study 2, using a daily diary study, replicated Study 1’s findings and further supported the mediation model at the within-person level. Considered together, these findings provide support for the effects of mindfulness, as a state and a trait, on alleviating depression and anxiety through increasing distress tolerance.

There are various theories regarding the mechanisms underlying mindfulness. One of the mechanisms consistently proposed by different researchers is exposure (Baer, 2003; Brown et al., 2007; Shapiro et al., 2006). Through exposure, individuals high in mindfulness tend to be aware of the aversive states without reacting to them automatically by avoiding or escaping (Treanor, 2011), and adopt a nonjudgmental stance towards emotional experiences (Lindsay & Creswell, 2017), which could lead to higher levels of distress tolerance (Kraemer et al., 2020). Distress tolerance is an important transdiagnostic factor underlying emotional disorders (for a review, see Leyro et al., 2010). However, until now, no study has directly examined whether distress tolerance is the underlying mechanism of the relationship between mindfulness and depression/anxiety.

The current study supported the mechanical role of distress tolerance at both between and within-person levels. Mindfulness and distress tolerance were mainly studied as an individual difference factor, less as a state that fluctuates across time and context within a person (Eisenlohr-Moul et al., 2016; Veilleux et al., 2018). However, Study 2 found that approximately half of the variance in mindfulness (ICC = .497) was at the within-person level, consistent with findings by Eisenlohr-Moul et al. (2016) (ICC = .46∼.56 for daily mindfulness subscales) and Hülsheger et al. (2013) (ICC = .62 for daily mindfulness). Regarding distress tolerance, Veilleux et al.'s study did not report the exact ICC for momentary distress tolerance but indicated its variability over time. The ICC of distress tolerance (.520) aligns with the findings of Doorley et al. (2019) study (ICC = .52). In addition, both anxiety and depression displayed a nontrivial amount of variability within persons (ICC = .452, .588).

Therefore, although these variables could be relatively stable, they also fluctuated over time within an individual. This opens the possibility to study their within-person relationships. Considered together with the results from study 1 and study 2, this suggests that mindful individuals experience less depression and anxiety due to higher levels of distress tolerance compared to less mindful individuals. On another important note, when an individual is more mindful on a given day than usual, they could better tolerate distress and have less depression/anxiety.

Apart from the concurrent relationship among mindfulness, distress tolerance, and depression/anxiety, the current study also examined their temporal precedence by establishing a time-lagged model. Results from Study 2 further supported that yesterday's mindfulness level could predict today's distress tolerance, which in turn predicts the next day's depression/anxiety. The mediating effect in the time-lagged model was significant, supporting the temporal precedence of mindfulness before distress tolerance. Alternative models in reverse order were also conducted (see Supplementary Materials). The significant mediating effect was nonsignificant at the between-person level in the time-lagged mediation model. Therefore, more evidence was found for the temporal precedence of mindfulness before distress tolerance, which is in line with studies that found intervening mindfulness could lead to increased distress tolerance (for a review, see Kramer et al., 2020). Since distress tolerance is strongly associated with and temporally precedes mindfulness, its role as a mediator has obtained rather solid evidence (Kazdin, 2007).

### Theoretical and Practical Implications

Theoretically, the current study is the first to provide evidence for distress tolerance, an important transdiagnostic factor of depression and anxiety, as the underlying mechanism of mindfulness. Although previous researchers consistently proposed that exposure is an important mechanism underlying mindfulness (e.g., Baer et al., 2003), no study has directly examined it to our knowledge. The current study broadened our understanding of the mechanical role of distress tolerance from both trait and state perspectives.

In practice, distress tolerance is a malleable factor that could be cultivated by certain interventions (Leyro et al., 2010). The two studies presented in this paper suggested that MBIs is a particularly promising and effective intervention for improving distress tolerance, which could in turn lead to reductions in depression/anxiety. In addition, since distress tolerance is a potential mechanism, interventions found to directly increase distress tolerance (e.g., interoceptive exposure; Barlow, 2010) could be incorporated into MBIs to enhance the efficacy of reducing depression/anxiety. Lastly, since distress tolerance mediates the relationship between mindfulness and depression/anxiety at both between-person and within-person levels, both long-term MBIs aimed at increasing trait mindfulness and short-term MBIs aimed at increasing state mindfulness can be beneficial for reducing depression/anxiety through increasing distress tolerance.

### Limitations and future research

Despite the important findings of this study, it is important to acknowledge the following limitations. Firstly, despite the demonstrated reliability of online assessment in generating data (Gwaltney et al., 2008), it is crucial to recognize its inherent limitations, including self-selection bias and potential issues with sample representativeness (Wright, 2006). Future studies should aim to address these limitations by implementing strategies (e.g., employing mixed-methods designs) to minimize self-selection bias and enhance sample representativeness.

Furthermore, although time-lagged models were also conducted in this study, it is important to note that the study design was inherently correlational. As a result, causal conclusions cannot be drawn from the current findings. Future studies should consider conducting randomized controlled trials that manipulate mindfulness or distress tolerance to examine the causal relationship between these variables and depression/anxiety.

Additionally, the absence of external variables, such as stressful circumstances or daily life experiences, in the models is another limitation of this study. Future research should address this limitation by incorporating these external variables to examine their impact on the proposed mediation model. This would provide a more comprehensive understanding of the complex relationships between mindfulness, distress tolerance, and anxiety/depression.

Lastly, while the daily diary study design employed in this study offers numerous advantages, such as providing rich temporal data, enhancing ecological validity, and allowing for the examination of within-person relationships, it is important to acknowledge that the shortening of the questionnaire may have contributed to the observed variability. Future studies should consider utilizing more comprehensive measures to further investigate these relationships.

## Author contribution statement

Yanjuan Li: Conceptualization, Fomal analysis, Investigation, Methodology, Writing-original draft, Writing-review & editing.

Ruilin Ju: Investigation, Validation

Stefan G. Hofmann: Writing-review & editing

Wingsze Chiu: Investigation

Ye Guan: Investigation

Yu Leng: Validation

Xinghua Liu: Conceptualization, Supervision, Writing-review & editing

## Declaration of Competing Interest

Dr. Hofmann receives financial support by the 10.13039/100003579Alexander von Humboldt Foundation (as part of the Alexander von Humboldt Professur), the 10.13039/501100003495Hessische Ministerium für Wissenschaft und Kunst (as part of the LOEWE Spitzenprofessur), NIH/NIMH R01MH128377, NIH/NIMHU01MH108168, Broderick Foundation/MIT, and the James S. McDonnell Foundation 21st Century Science Initiative in Understanding Human Cognition – Special Initiative. He receives compensation for his work as editor from SpringerNature. He also receives royalties and payments for his work from various publishers. Other authors declared no potential conflicts of interest with respect to the research, authorship, and/or publication of this article.

## Appendix Supplementary materials

Image, application 1

## References

[bib0001] Allen L.B., McHugh R.K., Barlow D.H., Barlow D.H. (2007). Clinical handbook of psychological disorders: A step-by-step treatment manual.

[bib0002] Baer R.A. (2003). Mindfulness training as a clinical intervention: A conceptual and empirical review. Clinical Psychology (New York, N.Y.).

[bib0003] Baer R.A., Smith G.T., Hopkins J., Krietemeyer J., Toney L. (2006). Using self–report assessment methods to explore facets of mindfulness. Assessment.

[bib0004] Barcaccia B., Baiocco R., Pozza A., Pallini S., Mancini F., Salvati M. (2019). The more you judge the worse you feel. A judgemental attitude towards one's inner experience predicts depression and anxiety. Personality and Individual Differences.

[bib0005] Barlow D.H., Farchione T.J., Fairholme C.P., Ellard K.K., Boisseau C.L., Allen L.B. (2010). Unified protocol for transdiagnostic treatment of emotional disorders. Politics.

[bib0006] Bentley K.H., Gallagher M.W., Carl J.R., Barlow D.H. (2014). Development and validation of the overall depression severity and impairment scale. Psychological Assessment.

[bib0007] Bishop S.R., Lau M., Shapiro S., Carlson L., Anderson N.D., Carmody J. (2004). Mindfulness: a proposed operational definition. Clinical Psychology: Science and Practice.

[bib0008] Bolger N., Laurenceau J.P. (2013).

[bib0009] Brem M.J., Khaddouma A., Elmquist J., Florimbio A.R., Shorey R.C., Stuart G.L. (2019). Relationships among dispositional mindfulness, distress Tolerance, and women's dating violence perpetration: A path analysis. Journal of Interpersonal Violence.

[bib0010] Brown K.W., Ryan R.M., Creswell J.D. (2007). Mindfulness: Theoretical foundations and evidence for its salutary effects. Psychological Inquiry.

[bib0011] Carpenter J.K., Sanford J., Hofmann S.G. (2019). The effect of a brief mindfulness training on distress tolerance and stress reactivity. Behavior Therapy.

[bib0012] Craske M.G., Kircanski K., Zelikowsky M., Mystkowski J., Chowdhury N., Baker A. (2008). Optimizing inhibitory learning during exposure therapy. Behaviour Research and Therapy.

[bib0013] Desrosiers A., Vine V., Klemanski D.H., Nolen-Hoeksema S. (2013). Mindfulness and emotion regulation in depression and anxiety: Common and distinct mechanisms of action. Depression and Anxiety.

[bib0014] Doorley J.D., Kashdan T.B., Alexander L.A., Blalock D.V., McKnight P.E. (2019). Distress tolerance in romantic relationships: A daily diary exploration with methodological considerations. Motivation and Emotion.

[bib0015] Eisenlohr-Moul T.A., Peters J.R., Pond R.S., DeWall C.N. (2016). Both trait and state mindfulness predict lower aggressiveness via anger rumination: A multilevel mediation analysis. Mindfulness.

[bib0016] Ellard K.K., Fairholme C.P., Boisseau C.L., Farchione T.J., Barlow D.H. (2010). Unified Protocol for the Transdiagnostic Treatment of Emotional Disorders: Protocol development and initial outcome data. Cognitive and Behavioral Practice.

[bib0017] Elhai J.D., Levine J.C., O'Brien K.D., Armour C. (2018). Distress tolerance and mindfulness mediate relations between depression and anxiety sensitivity with problematic smartphone use. Computers in Human Behavior.

[bib0018] Enkema M.C., McClain L., Bird E.R., Halvorson M.A., Larimer M.E. (2020). Associations between mindfulness and mental health outcomes: A systematic review of ecological momentary assessment research. Mindfulness.

[bib0019] Felsman P., Verduyn P., Ayduk O., Kross E. (2017). Being present: Focusing on the present predicts improvements in life satisfaction but not happiness. Emotion.

[bib0020] Germer C.K., Siegel R., Fulton P. (2005).

[bib0021] Gwaltney C.J., Shields A.L., Shiffman S. (2008). Equivalence of electronic and paper-and-pencil administration of patient-reported outcome measures: A meta-analytic review. Value in Health.

[bib0022] Hashoul-Andary R., Assayag-Nitzan Y., Yuval K., Aderka I.M., Litz B., Bernstein A. (2015). A longitudinal study of emotional distress intolerance and psychopathology following exposure to a potentially traumatic event in a community sample. Cognitive Therapy and Research.

[bib0023] Hawkins K.A., Macatee R.J., Guthrie W., Cougle J.R. (2013). Concurrent and prospective relations between distress tolerance, life stressors, and anger. Cognitive Therapy and Research.

[bib0024] Hou J., Wong S.Y., Lo H.H., Mak W.W., Ma H.S. (2014). Validation of a Chinese version of the Five Facet Mindfulness Questionnaire in Hong Kong and development of a short form. Assessment.

[bib0025] Hu L., Bentler P.M. (1999). Cutoff criteria for fit indexes in covariance structure analysis: Conventional criteria versus new alternatives. Structural Equation Modeling.

[bib0026] Hülsheger U.R., Alberts H.J., Feinholdt A., Lang J.W. (2013). Benefits of mindfulness at work: the role of mindfulness in emotion regulation, emotional exhaustion, and job satisfaction. The Journal of Applied Psychology.

[bib0027] Kass R.E., Raftery A.E. (1995). Bayes factors. Journal of the American Statistical Association.

[bib51] Kazdin A.E. (2007). Mediators and mechanisms of change in psychotherapy research. Annual Review of Clinical Psychology.

[bib0028] Kessler R.C., Andrews G., Colpe L.J., Hiripi E., Mroczek D.K., Normand S.L., Walters E.E., Zaslavsky A.M. (2002). Short screening scales to monitor population prevalences and trends in non-specific psychological distress. Psychological Medicine.

[bib0029] Kraemer K.M., Luberto C.M., Hall D.L., Ngo L.H., Yeh G.Y. (2020). A systematic review and meta-analysis of mindfulness- and acceptance-based interventions for affect intolerance/sensitivity. Behaviour Research and Therapy.

[bib0030] Lass A.N.S., Winer E.S. (2020). Distress tolerance and symptoms of depression: A review and integration of literatures. Clinical Psychology Science and Practice.

[bib0031] Leyro T.M., Zvolensky M.J., Bernstein A. (2010). Distress tolerance and psychopathological symptoms and disorders: a review of the empirical literature among adults. Psychological Bulletin.

[bib0032] Lin M.P., You J., Wu Y.W., Jiang Y. (2018). Depression mediates the relationship between distress tolerance and nonsuicidal self-injury among adolescents: One-year follow-up. Suicide and Life-Threat Behavior.

[bib0033] Liu, X. (in press). *Mindfulness Intervention for Emotional Distress.* Peking University Press.

[bib52] Lindsay E.K., Creswell J.D. (2017). Mechanisms of mindfulnesstraining: Monitor and acceptance theory (MAT). Clinical Psychology Review.

[bib0034] Lotan G., Tanay G., Bernstein A. (2013). Mindfulness and distress tolerance: Relations in a mindfulness preventive intervention. International Journal of Cognitive Therapy.

[bib0035] Lynch T.R., Mizon G.A., Zvolensky M.J., Bernstein A., Vujanovic A.A. (2011). Distress tolerance: Theory, research, and clinical applications.

[bib0036] Norman S.B., Campbell-Sills L., Hitchcock C.A., Sullivan S., Rochlin A., Wilkins K.C., Stein M.B. (2011). Psychometrics of a brief measure of anxiety to detect severity and impairment: the Overall Anxiety Severity and Impairment Scale (OASIS). Journal of Psychiatric Research.

[bib0037] Preacher K.J., Hayes A.F. (2004). SPSS and SAS procedures for estimating indirect effects in simple mediation models. Behavior Research Methods, Instruments, and Computers.

[bib0038] Preacher K.J., Selig J.P. (2012). Advantages of Monte Carlo confidence intervals for indirect effects. Communication Methods and Measures.

[bib0039] Preacher K.J., Zhang Z., Zyphur M.J. (2011). Alternative methods for assessing mediation in multilevel data: The advantages of multilevel SEM. Structural Equation Modeling.

[bib0040] Prieto-Fidalgo Á., Gómez-Odriozola J., Royuela-Colomer E., Orue I., Fernández-González L., Oñate L., Cortazar N., Iraurgi I., Calvete E. (2022). Predictive associations of dispositional mindfulness facets with anxiety and depression: A meta-analytic structural equation modeling approach. Mindfulness.

[bib0041] Reitzel L.R., Smith N.G., Obasi E.M., Forney M., Leventhal A.M. (2017). Perceived distress tolerance accounts for the covariance between discrimination experiences and anxiety symptoms among sexual minority adults. Journal of Anxiety Disorders.

[bib0042] Sauer S., Baer R.A, Baer R.A. (2010). Assessing mindfulness and acceptance processes in clients.

[bib0043] Shapiro S.L., Carlson L.E., Astin J.A., Freedman B. (2006). Mechanisms of mindfulness. Journal of Clinical Psychology.

[bib0044] Simons J., Gaher R. (2005). The Distress Tolerance Scale: Development and validation of a self-report measure. Motivation and Emotion.

[bib0045] Taylor H., Strauss C., Cavanagh K. (2021). Can a little bit of mindfulness do you good? A systematic review and meta-analyses of unguided mindfulness-based self-help interventions. Clinical Psychology Review.

[bib50] Trafton J.A., Gifford E.V., Zvolensky M.J., Bernstein A., Vujanovic A.A. (2011). *Distress Tolerance: Theory, Research, and Clinical Application*.

[bib0046] Treanor M. (2011). The potential impact of mindfulness on exposure and extinction learning in anxiety disorders. Clinical Psychology Review.

[bib0047] Veilleux J.C., Hill M.A., Skinner K.D., Pollert G.A., Baker D.E., Spero K.D. (2018). The dynamics of persisting through distress: Development of a momentary distress intolerance scale using ecological momentary assessment. Psychological Assessment.

[bib0048] Wright K.B. (2006). Researching Internet-Based populations: Advantages and disadvantages of online survey research, online questionnaire authoring software packages, and web survey services. Journal of Computer-Mediated Communication.

[bib0049] Yoon S., Dang V., Mertz J., Rottenberg J. (2018). Are attitudes towards emotions associated with depression? A conceptual and meta-analytic review. Journal of Affective Disorders.

