# Supplementary material for: Distress tolerance as a mechanism of mindfulness for depression and anxiety: Cross-sectional and diary evidence
Source: Int J Clin Health Psychol. 2023 Jul 5;23(4):100392. doi: 10.1016/j.ijchp.2023.100392 (PMC10345371; doi:10.1016/j.ijchp.2023.100392)
Supplement: Supplementary file 1 [file mmc1.docx]

**SUPPLEMENTARY MATERIALS**

Study 1

Reversed mediation models with distress tolerance as the predictor and mindfulness as the mediator were also conducted. As shown in Fig S1, after controlling for age, distress tolerance positively predicted mindfulness and negatively predicted depression/anxiety, and mindfulness negatively predicted depression/anxiety. In addition, the paths from distress tolerance through mindfulness to both depression (*a***b*_1_ = -0.036, *SE* = 0.006, 95%CI = [-0.048, -0.023]) and anxiety (*a***b*_2_ = -0.035, *SE* = 0.007, 95%CI = [-0.048, -0.019]) were statistically significant. The ratios of the indirect effect to the total effect were 19.67% and 20.23% respectively. The model fit the data well (AIC = 22025, BIC = 22102, $\chi_{\left( 2 \right)}^{2}$ = 15.441, *p* < .001, CFI = 0.990, RMSEA = 0.086, 90%CI = [0.050, 0.128], SRMR = 0.039). These findings suggest that mindfulness partially mediated the effects of distress tolerance on both depression and anxiety.


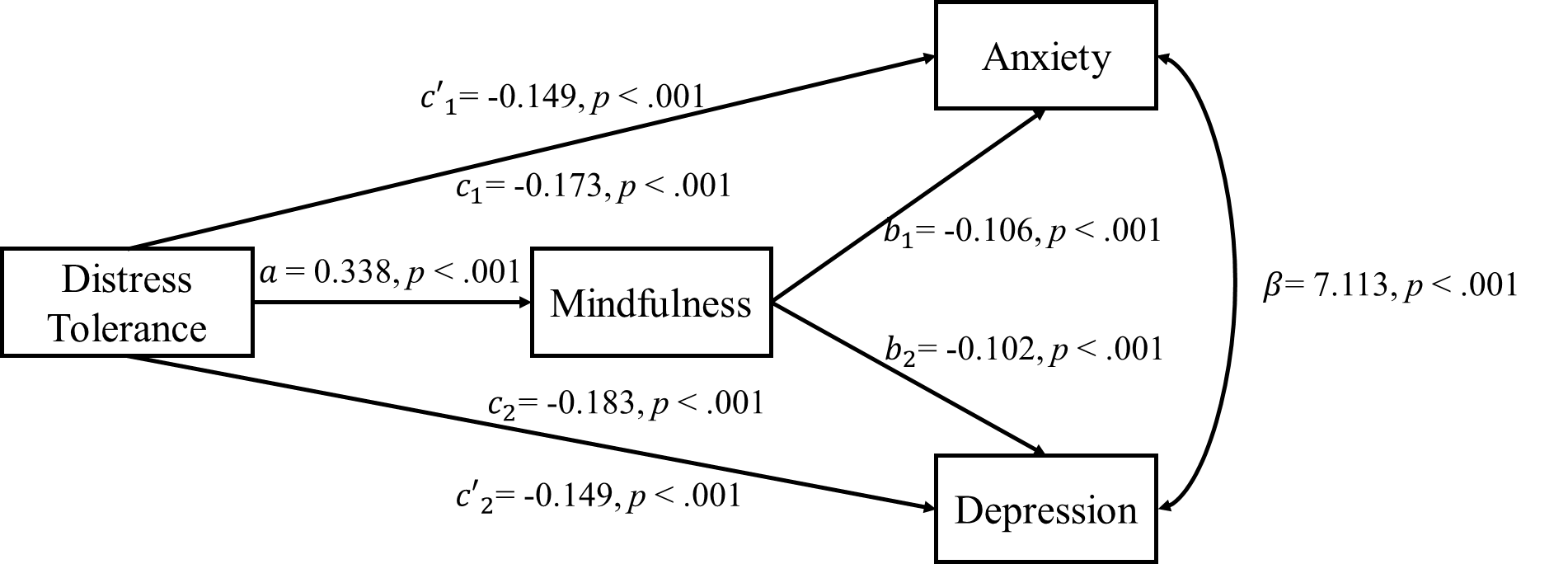


**Fig S**1. Path diagram of the reversed mediation model in which mindfulness was assumed to mediate the relationship between distress tolerance and depression/anxiety of Study 1.

Study 2

Reversed mediation models with daily distress tolerance as the predictor and daily mindfulness as the mediator were also conducted. For the concurrent mediation model, the indirect effects of daily mindfulness in the relationship between distress tolerance and depression/anxiety were significant at the within-person level but not the between-person level (See Fig S2 and Table S1). The model fit the data well (AIC = 25486, BIC = 25622, $\chi_{\left( 2 \right)}^{2}$ = 16.050, *p* < .001, CFI = 0.986, RMSEA = 0.072, within-level SRMR = 0, between-level SRMR = 0.074).

For the time-lagged mediation model, the indirect effects of daily mindfulness were significant at the within-person level but not the between-person level (See Fig S3 and Table S1). The model fit the data well (AIC = 24791, BIC = 24930, $\chi_{\left( 2 \right)}^{2}$ = 15.652, *p* < .001, CFI = 0.975, RMSEA = 0.067, within-level SRMR = 0, between-level SRMR = 0.074).


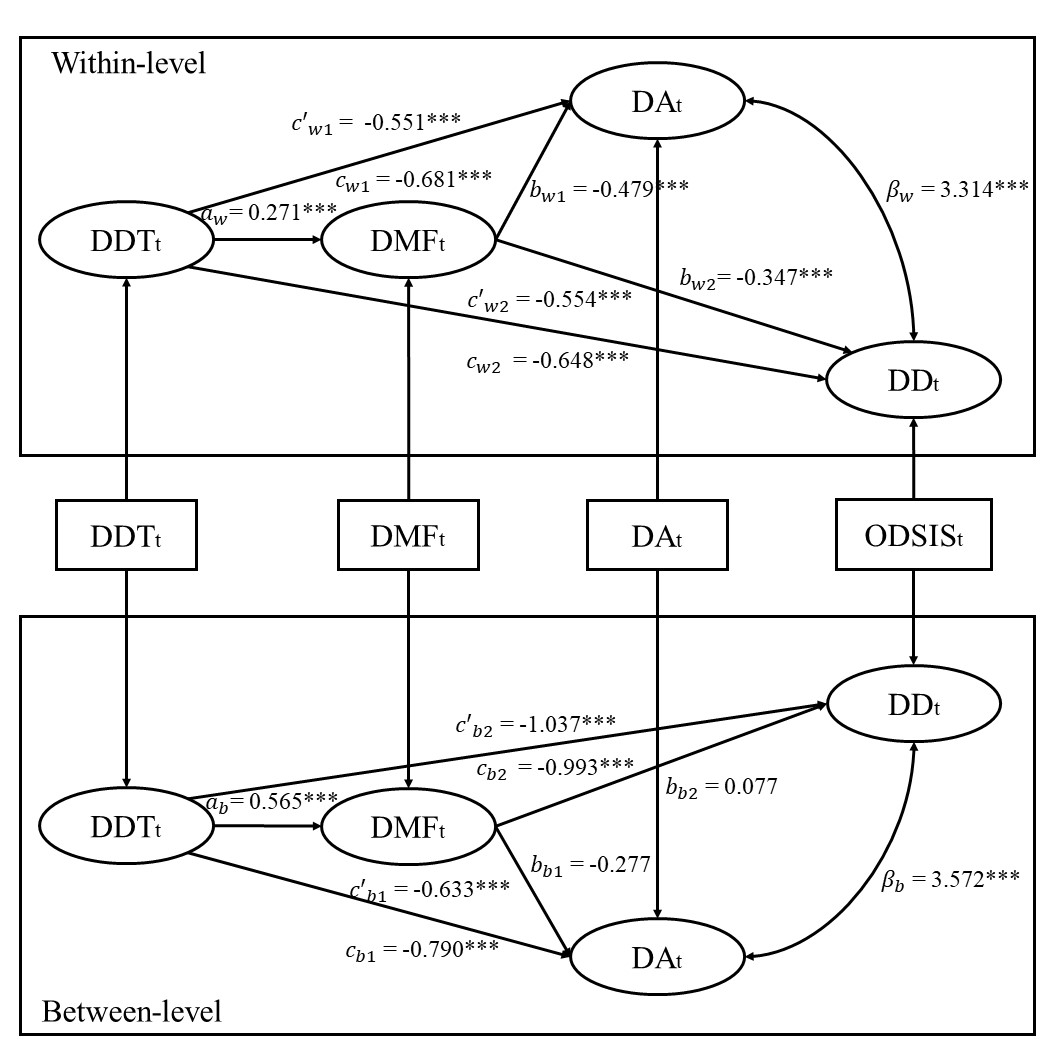


Fig S2. MSEM results for the 1-1-1 concurrent mediation model of DDTto DA/DD via DMF. ****p* < .001, ***p* < .01, **p* < .05; DMF = daily mindfulness；DDT = daily distress tolerance; DA = daily anxiety; DD = daily depression.


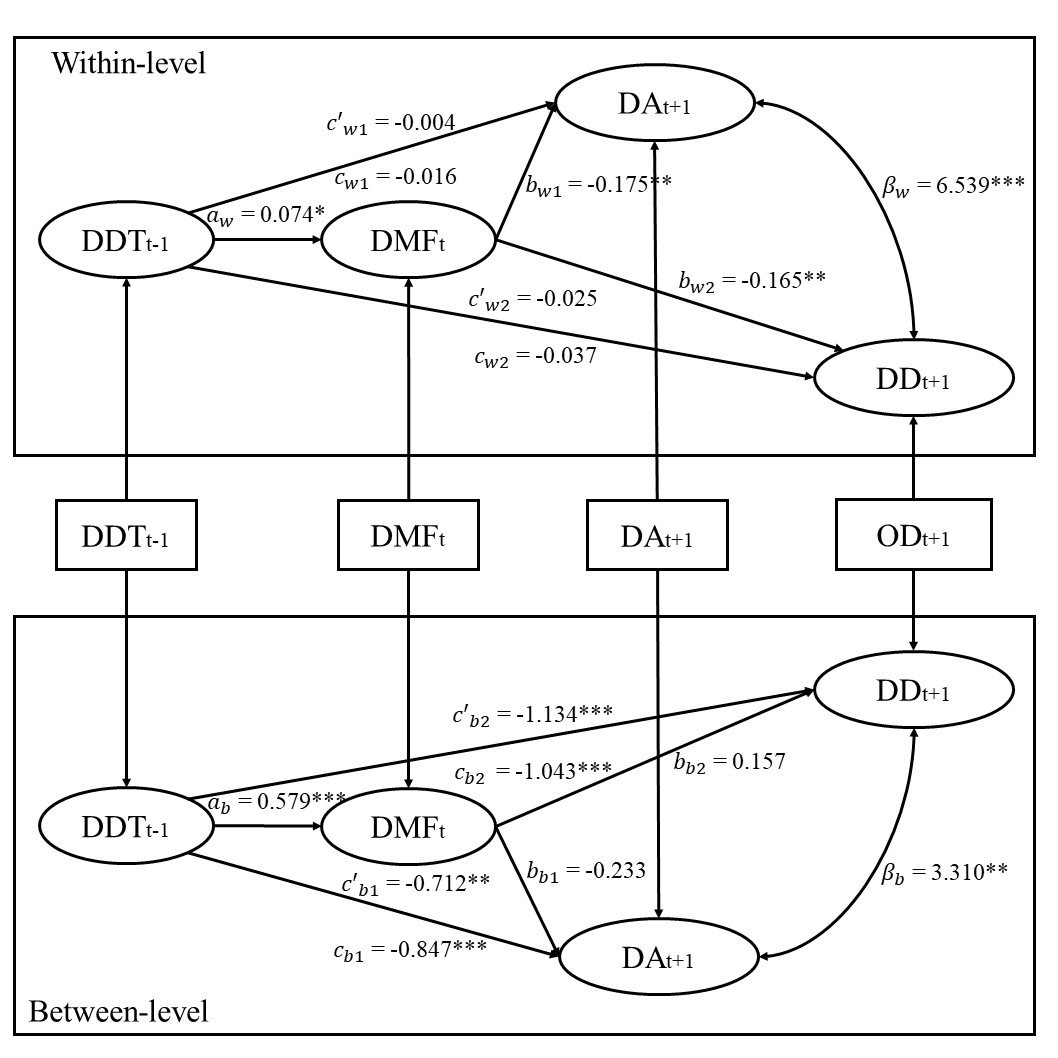


Fig S3. MSEM results for the 1-1-1 time-lagged mediation model of DDT at t-1 to DA/DD at t+1 via DMF at t. ****p* < .001, ***p* < .01, **p* < .05; DMF = daily mindfulness；DDT = daily distress tolerance; DA = daily anxiety; DD = daily depression.

Table S1. Indirect effects of the multilevel mediation model of daily mindfulness as the mediator

|  |  | **Indirect Effects** | ***SE*** | **95% MCCI** |
| --- | --- | --- | --- | --- |
| $\mathrm{DDT}_{t}$ 🡪 $\mathrm{DMF}_{t}$ 🡪 $\mathrm{DA}_{t}$/$\mathrm{DD}_{t}$ | | |  |  |
| Within-level | *a_b_***b_b_*_1_ | -0.130 | 0.020 | [-0.172, -0.092] |
|  | *a_b_***b_b_*_2_ | -0.094 | 0.021 | [-0.136, -0.056] |
| Between-level | *a_b_***b_b_*_1_ | -0.157 | 0.136 | [-0.414, 0.125] |
|  | *a_b_***b_b_*_2_ | 0.043 | 0.209 | [-0.324, 0.507] |
| $\mathrm{DDT}_{t-1}$ 🡪 $\mathrm{DMF}_{t}$ 🡪 $\mathrm{DA}_{t+1}$/$\mathrm{DD}_{t+1}$ | | |  |  |
| Within-level | *a_b_***b_b_*_1_ | -0.013 | 0.007 | [-0.028, -0.002] |
|  | *a_b_***b_b_*_2_ | -0.012 | 0.006 | [-0.026, -0.002] |
| Between-level | *a_b_***b_b_*_1_ | -0.135 | 0.159 | [-0.428, 0.202] |
|  | *a_b_***b_b_*_2_ | 0.091 | 0.247 | [-0.336, 0.637] |

*Note. SE* = standard error, MCCI = Monte Carlo Confidence Interval. DDT = daily distress tolerance; DMF = daily mindfulness; DA = daily anxiety; DD = daily depression.
